# Cerebrospinal fluid immunoglobulin light chain ratios predict disease progression in multiple sclerosis

**DOI:** 10.1136/jnnp-2018-317947

**Published:** 2018-05-09

**Authors:** Emma Rathbone, Lindsay Durant, James Kinsella, Antony R Parker, Ghaniah Hassan-Smith, Michael R Douglas, S John Curnow

**Affiliations:** 1 Centre for Translational Inflammation Research Institute of Inflammation and Ageing, College of Medical and Dental Sciences University of Birmingham, Birmingham, UK; 2 The Binding Site Group Ltd, Birmingham, UK; 3 Department of Neurology, Dudley Group NHS Foundation Trust, Russells Hall Hospital, Birmingham, UK; 4 School of Life and Health Sciences, Aston University, Birmingham, UK

## Abstract

**Objective:**

To determine whether the ratio of cerebrospinal fluid (CSF) immunoglobulin kappa to lambda light chains at time of multiple sclerosis (MS) diagnosis predicts disease progression and whether this was intrinsic to CSF plasmablasts.

**Methods:**

CSF and peripheral blood were obtained from patients undergoing elective diagnostic lumbar puncture and included clinically isolated syndrome (CIS) (n=43), relapsing remitting MS (RRMS; n=50), primary progressive MS (PPMS; n=20) and other neurological disease controls, both inflammatory (ONID; n=23) and non-inflammatory (OND; n=114). CSF samples were assayed for free and immunoglobulin-associated light chains and on B cells and plasmablasts. Clinical follow-up data were collected during a 5-year follow-up period where available.

**Results:**

There was an increased median CSF κ:λ free light chain (FLC) in all MS groups (CIS: 18.2, 95% CI 6.8 to 30.3; RRMS: 4.4, 95% CI 2.7 to 11.4; PPMS: 12.0, 95% CI 3.6 to 37.1) but not controls (OND: 1.61, 95% CI 1.4 to 1.9; ONID: 1.7, 95% CI 1.3 to 2.2; p<0.001). This ratio predicted Expanded Disability Status Scores (EDSS) progression at 5 years, with a lower median EDSS in the group with high (>10) CSF κ:λ FLC (0.0, 95% CI 0 to 2.5 vs 2.5, 95% CI 0 to 4, high vs low; p=0.049). CSF κ:λ FLC correlated with CSF IgG1 κ:λ (r=0.776; p<0.0001) and was intrinsic to CSF plasmablasts (r=0.65; p=0.026).

**Conclusions:**

These data demonstrate that CSF immunoglobulin κ:λ ratios, determined at the time of diagnostic lumbar puncture, predict MS disease progression and may therefore be useful prognostic markers for early therapeutic stratification.

## Introduction

Multiple sclerosis (MS) is a chronic inflammatory autoimmune disease of the central nervous system, usually presenting as a clinically isolated syndrome (CIS). Not all patients convert to clinically definite MS, and in those who do the level of disability accumulation is highly variable.^1^ Validated long-term prognostic markers are therefore important in guiding the therapeutic choices for individual patients. Clinical indicators of poor prognosis include a short-time interval between the first two relapses, rapid evolution of disease and older age at onset.^2 3^ Baseline MRI lesional topography provides some information, and infratentorial lesions and/or spinal cord lesions have some predictive value for disability accumulation.^4 5^ The best-established cerebrospinal fluid (CSF)-based diagnostic biomarker are oligoclonal bands (OCB), with a potential role in prognostication,^6^ although this observation is not consistent.^7^ OCB analyses have key issues with reproducibility and, as a qualitative assay, a limited dynamic range. Other CSF markers have been linked to disease progression including neurofilament light chains (NFL) and chitinase-3-like 1 (CHI3L1).^8 9^ In a manner analogous to OCB, we and others have used CSF immunoglobulin (Ig) free light chains (FLC) as biomarkers for confirming a diagnosis of MS, showing greater sensitivity and specificity than OCB.^10^ There is a general excess of κ over λ FLC,^10–12^ and in one recent study, the CSF κ:λ FLC ratio was shown to predict conversion from CIS to clinically definite MS (CDMS).^13^ In this study, we have examined the utility of the CSF FLC κ:λ ratio as a marker for the development of MS disability.

## Methods

### Standard protocol approvals, registrations and patient consents

All subjects provided written informed consent, in accordance with the Declaration of Helsinki and were not on disease-modifying therapies at the time of sampling and had not received steroids for at least 3 months prior to sampling.

### Experimental design

Matched peripheral blood and CSF samples were prospectively collected during routine diagnostic lumbar puncture, and blinding was achieved, with all samples analysed before the diagnosis had been formally determined. Samples were collected from patients with MS fulfilling 2010 revisions of the McDonald criteria,^14^ classified as CIS, relapsing remitting MS (RRMS) or primary progressive MS (PPMS), as well as patients with other neurological diseases (OND) and other neurological inflammatory diseases (ONID) (table 1). Expanded Disability Status Scores (EDSS) at 5 years following the diagnostic lumbar puncture, annualised relapse rates during that period, use of first-line and second-line therapy and conversion from CIS to CDMS were obtained where available. All diagnostic MRI series were further reviewed, with categorisation of infratentorial lesions, according to Minneboo *et al.* 5 During clinical follow-up, patients were excluded where (1) no definitive follow-up data were available, (2) the CSF samples were OCB negative and/or (3) where the FLC values were below the detection limit, not permitting evaluation of an accurate CSF FLC κ:λ ratio (online Supplementary figure e1).

10.1136/jnnp-2018-317947.supp1 Supplementary file 1

**Table 1:** Characteristics of cohorts used for figure 1

Disease group	Age*	Gender (F:M)
CIS† (n=43)	45.0 (21–70)	2.73:1
RRMS‡ (n=50)	40.5 (19–72)	3.00:1
PPMS§ (n=20)	54.5 (35–73)	1.86:1
OND¶ (n=114)	46.0 (17–85)	2.08:1
ONID** (n=23)	55.0 (24–83)	0.91:1

*Median (range).

†Clinically isolated syndrome.

‡Relapsing-remitting multiple sclerosis.

§Primary-progressive multiple sclerosis.

¶Other neurological diseases.

**Other neurological inflammatory diseases.

### Preparation of CSF and peripheral blood

CSF was obtained by non-traumatic lumbar puncture and centrifuged (600 *g*, 8 min). The cell-free CSF was collected and stored at −80°C, and CSF cells were stained for flow cytometry (see below). Peripheral blood was collected into EDTA-containing tubes (Greiner Bio-One, Gloucestershire, UK) and peripheral blood mononuclear cells (PBMC) isolated by density gradient centrifugation (Ficoll-Paque Plus; GE Healthcare Bioscience).

### Flow cytometric analysis

PBMC and CSF cells were analysed as detailed in the online Supplementary figure e2.

### FLC quantification

FLC concentrations were measured by nephelometry using the latex particle-enhanced, Freelite κ and λ immunoassays^15^ on a Dade-Behring BN II Analyser, following the manufacturer’s instructions (The Binding Site Group Ltd, Birmingham, UK); assays were performed by The Binding Site Group Ltd, using anonymised coded samples. Normal serum reference intervals were used: κ FLC 3.3–19.4 mg/L and λ FLC 5.7–26.3 mg/L with an assay sensitivity of <1 mg/L.^16^ Normal CSF reference intervals have not been defined. The limit of detection for the κ FLC assay was 0.06 mg/L and 0.05 mg/L for the λ FLC assay.

### CSF NFL quantification

CSF NFL were measured by ELISA according to the manufacturer’s instructions (Uman Diagnostics, Umeå, Sweden). CSF samples were diluted 1:1 with sample diluent buffer and concentrations calculated from a standard curve.

### Ig heavy and light chain isotype determination

Antibodies specific for human IgA, IgG1-Fc, IgG2-Fc, IgG3 hinge, IgG4-Fc and IgM (Southern Biotech, Birmingham, Alabama, USA) were coupled to carboxylated xMAP fluorescent microspheres using the xMAP Antibody Coupling Kit (Luminex Corporation, Austin, Texas, USA) according to the manufacturer’s instructions. For CSF analysis, all incubation steps were for 30 min at room temperature with agitation followed by two washes in phosphate-buffered saline (PBS). CSF samples (50 µL) were added to a mixture of antibody-coated microspheres (50 µL) and 50 µL assay buffer (PBS 5%, bovine serum albumin, 5% goat serum), followed by 50 µL of biotin goat anti-human κ or λ light chain-specific antibodies (Southern Biotech; 2 µg/mL in assay buffer), then streptavidin R-phycoerythrin (Biolegend; 4 µg/mL). Samples were analysed using a Luminex-200 and xMAP software, with final concentrations calculated from standard curves.

### IGKV and IGLV sequencing

RNA isolation and cDNA synthesis from CSF cell pellets were performed using the μMACS One-step cDNA Kit (Miltenyi Biotec) according to the manufacturer’s instructions. Synthesised cDNA was eluted from the column and collected with 50 µL cDNA elution buffer. Immunoglobulin Kappa Variable (IGKV) and Immunoglobulin Lambda Variable (IGLV) sequencing of cDNA were performed by Adaptive Biotechnologies (Seattle, Washington, USA) using the immunoSEQ survey level assay.

### Data analysis

Statistical analyses were performed using GraphPad Prism V.7.0 (GraphPad Software, California, USA). All data sets were determined to require non-parametric testing following use of the D’Agostino and Pearson normality test and were two tailed.

## Results

### Elevated CSF κ:λ Ig light chain ratios are present in MS

**Figure 1:**
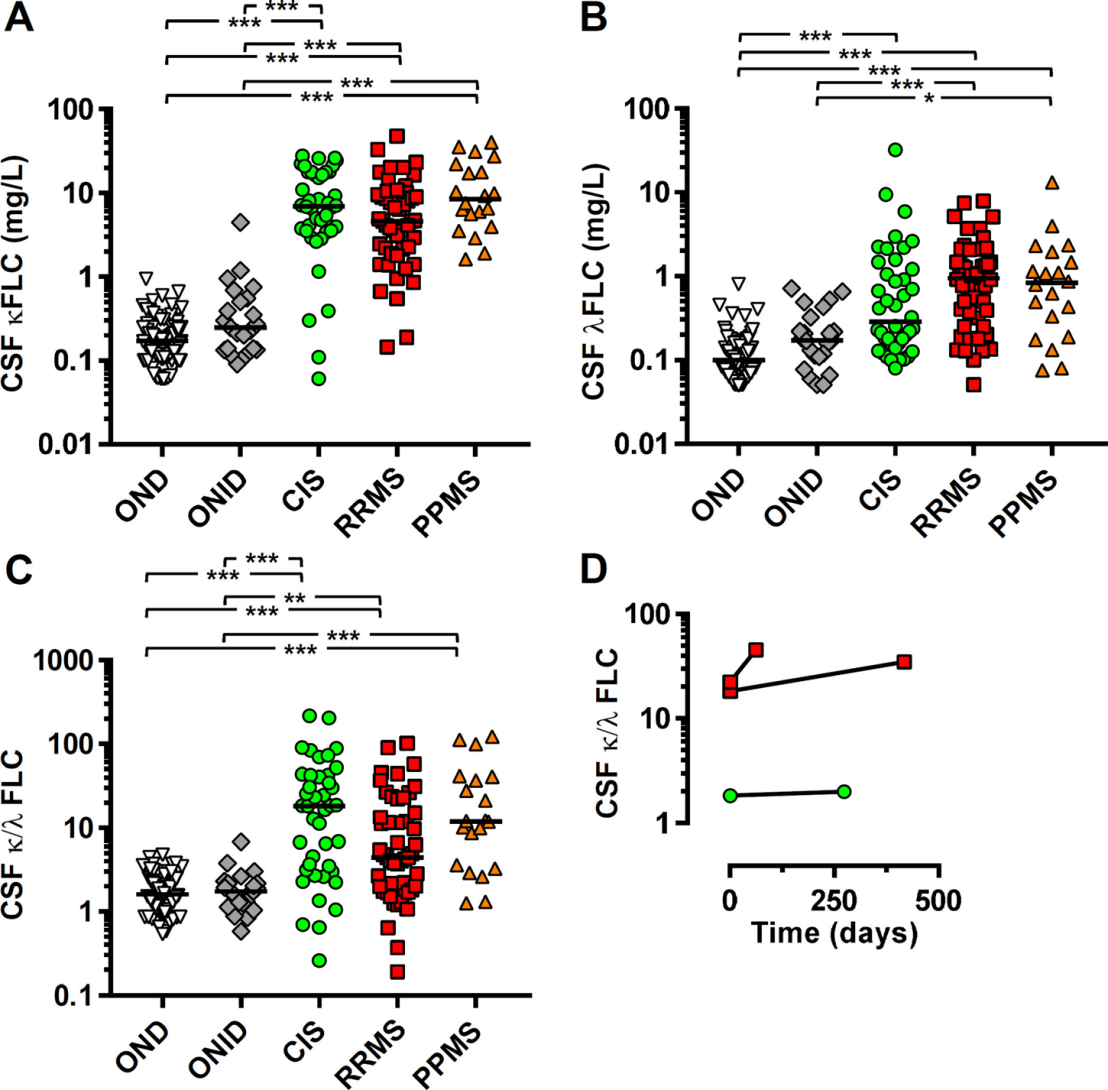
Highly elevated cerebrospinal fluid (CSF) free light chains (FLC) κ:λ ratios are detectable in multiple sclerosis (MS). The concentrations of (A) κ and (B) λ FLC and (C) the ratio were determined for CSF from clinically isolated syndrome (CIS), relapsing-remitting MS (RRMS), primary-progressive MS (PPMS), other neurological diseases (OND) and other neurological inflammatory diseases (ONID). (D) Repeat CSF samples at the indicated time interval were also analysed where available for one CIS (green circle) and two RRMS (red squares). Kruskal-Wallis with Dunn’s post-test; *p<0.05; **p<0.01; ***p<0.001, all other comparisons non-significant (p>0.05).

Ig FLC, in addition to OCB, are present in the CSF of patients with MS and are highly sensitive and specific in supporting a diagnosis of MS.^10 17 18^ Here, we confirm these findings in a large cohort, demonstrating elevated κFLC, and to a lesser extent λFLC, in the majority of patients with CIS, RRMS and PPMS (figure 1A, B). The lower λFLC levels led us to examine the ratio of CSF κ:λ FLC, recently suggested to be elevated in some MS CSF and be prognostic for CIS to CDMS conversion.^11^ Within all MS groups, we observed significantly elevated but highly variable CSF κ:λ FLC ratios, but not in control groups (figure 1C); CIS 18.2; 95% CI 6.8 to 30.3, RRMS 4.4; 95% CI 2.7 to 11.4, PPMS 12.0; 95% CI 3.6 to 37.1, OND 1.61; 95% CI 1.4 to 1.9 and ONID 1.7; 95% CI 1.3 to 2.2. Repeated CSF analysis in three individuals with RRMS showed a largely stable ratio over an interval of up to 418 days (figure 1D). The bias towards κFLC was not observed in the serum of these individuals (data not shown).

### CSF κ:λ FLC ratios predict MS disability accumulation

**Figure 2:**
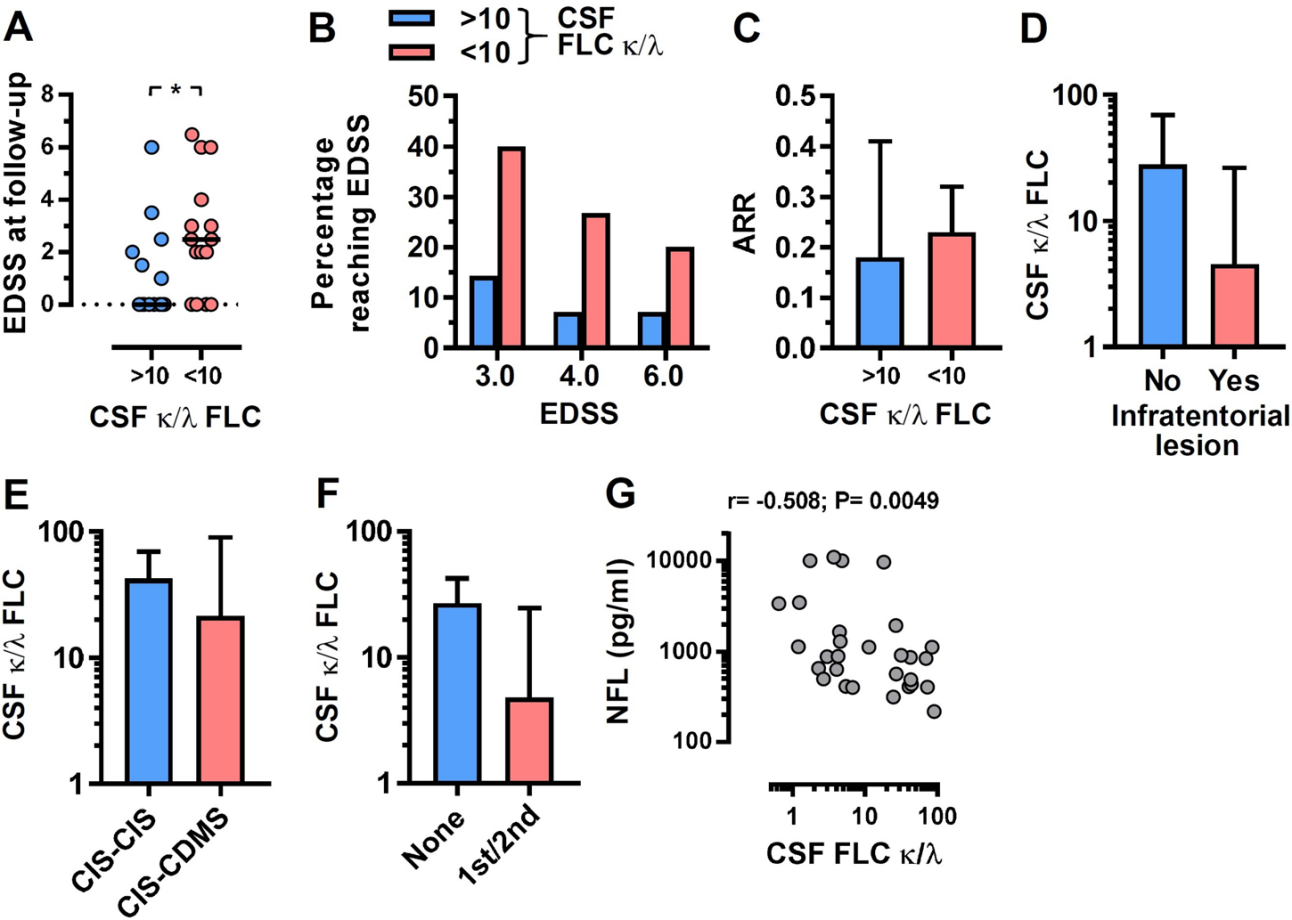
Cerebrospinal fluid (CSF) κ:λ free light chain (FLC) ratio predicts disability accumulation in multiple sclerosis (MS). (A) Expanded disability status scale (EDSS) at 5-year follow-up (following diagnostic lumbar puncture; where available) for high (>10) and low (<10) CSF FLC κ:λ ratio groups (n=15 and n=14, respectively); Mann-Whitney test; *p<0.05. The percentage of individuals reaching (B) EDSS 3.0, 4.0 or 6.0, (C) annualised relapse rates (ARR), (D) the presence of infratentorial lesions, (E) those remaining at clinically isolated syndrome (CIS) (CIS–CIS) or converting to clinically definite MS (CIS–CDMS) and (F) those requiring physician determined first-line or second-line therapy are shown for the high and low CSF κ:λ ratio groups. (G) Correlation between CSF neurofilament light chains and immunoglobulin FLC κ:λ ratios; Spearman correlation.

The highly variable CSF κ:λ FLC ratio in all MS groups led us to assess whether this ratio might be prognostic for disability accumulation. EDSS were determined at a 5-year follow-up time point following the diagnostic lumbar puncture. We divided the cohort into high (>10) and low (<10) CSF κ:λ FLC ratio groups based on approximately equal numbers in each group. There was a significantly lower EDSS at follow-up in the group of individuals with a high CSF κ:λ FLC ratio, with the lower ratio group accumulating greater disability (figure 2A). There was also a significant correlation between CSF κ:λ FLC ratio and EDSS at follow-up (r=−0.37; p=0.049). The sensitivity and specificity of a CSF κ:λ FLC ratio at <9.0 for predicting an EDSS of ≥3.0 at the 5-year follow-up period was 75.0% and 57.1%, respectively. Neither kappa nor lambda by themselves were significantly associated with disability progression. For EDSS 3.0, 4.0 and 6.0, there were greater proportions reaching these scores during the follow-up period in the lower CSF κ:λ FLC ratio group (figure 2B). We also examined several other standard clinical disease activity-related parameters. Although none of these reached statistical significance, likely due to the number of individuals where sufficiently detailed clinical data were available, all were consistent with the CSF κ:λ FLC ratio predicting disease severity. There was a small increase in annualised relapse rates over the 5-year follow-up period in the low CSF κ:λ FLC ratio group (figure 2C; 0.18 vs 0.23), a lower CSF κ:λ FLC ratio in those with infratentorial lesions (figure 2D), a higher κ:λ FLC ratio at diagnostic lumbar puncture for the group that remained CIS at the end of a 5-year follow-up period (figure 2E; 42.4 vs 21.4) and the group that required neither first-line or second-line therapy during the 5-year follow-up period, as determined by the treating physician, had a higher CSF κ:λ FLC ratio (figure 2F; 26.9 vs 4.8). There was no correlation between the CSF κ:λ FLC ratio and the time between disease onset and FLC measurement (r=−0.1207; p=0.56). Collectively, these data show that the CSF κ:λ FLC ratio at the time of diagnosis is predictive of the subsequent MS disease course. Concentrations of NFL in CSF have been previously associated with disease progression in MS.^19^ Consistent with this observation, there was a negative correlation between CSF NFL and Ig κ:λ FLC ratio (figure 2F). However, CSF NFL levels did not significantly correlate with disability accumulation (r=0.16; p=0.42) indicating that the CSF κ:λ FLC ratio is a better biomarker in MS.

### Altered Ig light chain ratios are intrinsic to CSF plasmablasts

**Figure 3:**
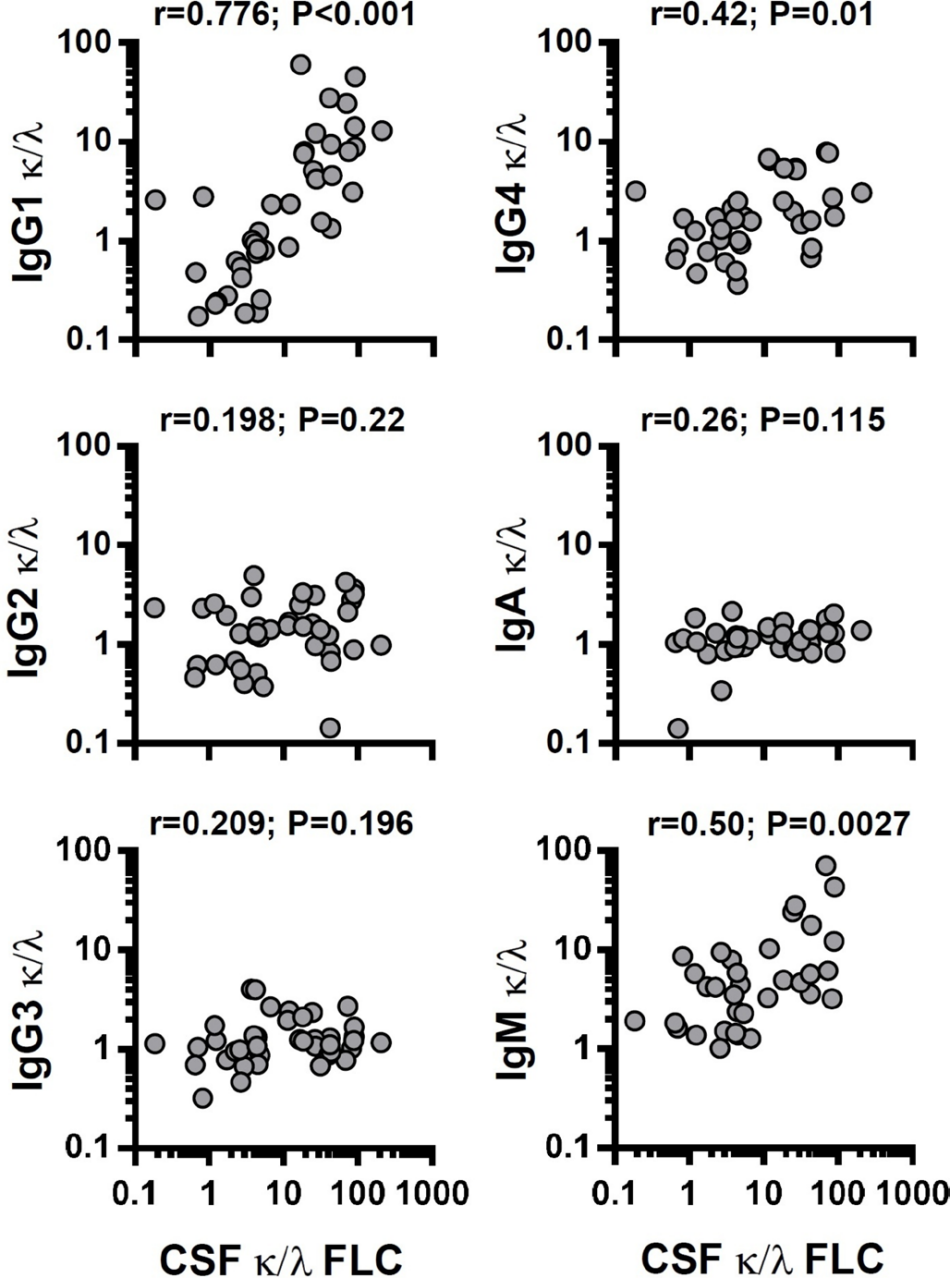
Correlation between cerebrospinal fluid (CSF) free light chain (FLC) and intact immunoglobulin κ:λ ratios. Correlations are shown between CSF FLC κ:λ ratio and the κ:λ ratio of CSF immunoglobulin (Ig)G1, IgG2, IgG3, IgG4 and IgM; Spearman correlations.

**Figure 4:**
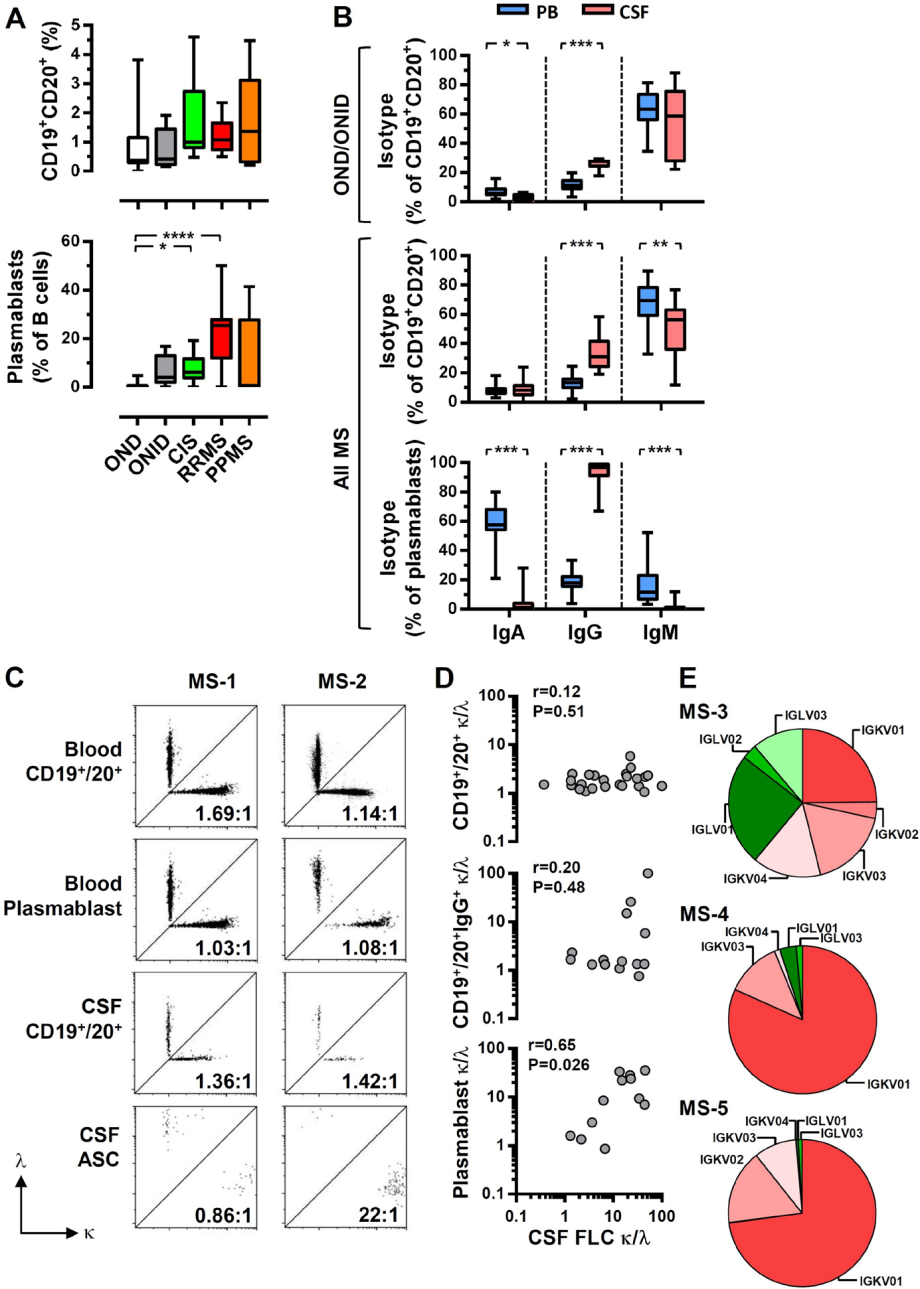
The bias to κ light chain usage is intrinsic to cerebrospinal fluid (CSF) plasmablasts. (A) The frequency of B cells (CD19+ and CD20+) and plasmablasts (according to the gating strategy in the online Supplementary figure e1) was determined for clinically isolated syndrome (CIS), relapsing-remitting multiple sclerosis (RRMS), primary-progressive multiple sclerosis (PPMS), other neurological diseases (OND) and other neurological inflammatory diseases (ONID). (B) For CSF and matched peripheral blood samples, immunoglobulin (Ig)A, IgG and IgM isotypes were determined on both CD19+ CD20+ B cells and plasmablasts. Kruskal-Wallis with Dunn’s post-test; ***p<0.001; **p<0.01; *p<0.05, all other comparisons non-significant (p>0.05). The frequency of κ-expressing and λ-expressing total B cells, IgG+ B cells and plasmablasts was determined by flow cytometry (C; representative plots for CIS (MS1) and RRMS (MS2)) and correlated with the CSF free light chain (FLC) κ:λ ratio (D); Spearman correlations. (E) The frequency of each Immunoglobulin Kappa Variable (IGKV); red and Immunoglobulin Lambda Variable (IGLV); green) gene segments was determined from the cDNA of CSF cells for MS3 (PPMS), MS4 (CIS) and MS5 (CIS).

There are several possible explanations for the bias towards κFLC in the CSF of patients with MS, unrelated to their antigenic specificity, including assay-dependent sensitivity for either κ or λ, in the FLC and/or intact Ig. We therefore examined the κ and λ light chains present in the intact IgG1, IgG2, IgG3, IgG4, IgA and IgM in CSF of patients with MS. It was clear that the observed bias towards CSF κFLC was recapitulated when examining IgG1κ and IgG1λ, with a strong correlation between the IgG1 κ:λ ratio and FLC κ:λ ratio (figure 3). There were also significant, but far weaker, correlations with IgG4 and IgM. To formally establish that the CSF FLC κ:λ ratio is directly associated with intrathecal antibody-secreting cells, we examined CSF B cells and plasmablasts. There were few B cells in the CSF of the control cohorts (OND and ONID), which increased in MS groups, although not reaching statistical significance (figure 4A, Supplementary figure e2). In contrast, there were significant increases in both CIS and RRMS plasmablasts, with none detected in the OND cohort (figure 4A), consistent with previous reports.^20^ There were significant increases in IgG+ B cells in the CSF as compared with blood, suggesting exclusion of IgA+ B cells (figure 4B, online Supplementary figure e1). Strikingly, CSF plasmablasts were almost entirely IgG+, consistent with the high levels of IgG1 detected in the CSF. Peripheral blood B cell and plasmablast κ:λ ratios were normal, whereas in some CSF samples, there was a clear bias towards κ-expressing plasmablasts (figure 4C), with the CSF κ:λ FLC ratio significantly correlating with the plasmablast but not the CD19+ CD20+ B cell κ:λ FLC ratios (figure 4D). To further validate that the high CSF κ:λ FLC ratios were intrinsic to the CSF cells, we sequenced the CDR3 Ig light chain regions of the CSF cells from three patients with MS (figure 4E), with use of cDNA biasing towards plasmablast-derived sequences.^21^ All samples showed oligoclonal populations as determined by both unique clone identification and gene segment usage. One showed little bias to IGKV, whereas the other two demonstrated a dramatic bias towards IGKV, with very few IGLV sequences detected. In summary, the results demonstrate that the high CSF κ:λ FLC ratios, which predict disability accumulation, are not an epiphenomenon of FLC measurement but result from a selective expansion of κ light chain expressing plasmablasts found within the CSF of patients with MS.

## Discussion

Although the past two decades have seen major advances in the sophistication and availability of diagnostic modalities used in MS (particularly MRI), the prognostic heterogeneity of MS makes temporal disability prediction extremely difficult for any given individual.^22^ The most recent MRI in multiple sclerosis consensus guidelines highlight the prognostic limitations of MRI in the prediction of disability^23^ and validated prognostic markers are critically needed. The presence of CSF OCB at diagnosis doubles the risk of further relapse, but does not provide further information on the development of disability.^24^ The detection of IgM OCB may be an exception, with positive patients experiencing more relapses, a higher requirement for therapy and accumulation of more disability.^25^

In this study, we demonstrated, for the first time, that the ratio of κ:λ Ig FLC measured at the time of diagnostic lumbar puncture predicts EDSS disability at 5 years postdiagnosis. This is a critical advance on previous studies indicating that the ratio of κ:λ FLC in the CSF might predict the conversion from CIS to CDMS.^11^ The strength of the correlation was not very high (r=−0.37) suggesting that additional factors influence the accumulation of disability. Although we did not detect a significant difference when examining conversion from CIS, it was clear that patients with MS demonstrating a high CSF κ:λ FLC were more likely to remain CIS over time. Voortman *et al* failed to detect any relationship to disability progression, possibly due to a lower frequency of high CSF κ:λ FLC ratio individuals in their study and/or the shorter follow-up period examined. Our data show that for patients with MS demonstrating a low CSF κ:λ light chain ratio at diagnosis, the combined profile of greater conversion from CIS, higher relapse rates, increased disability progression and greater requirement for therapeutic intervention suggest that this immunological feature of MS is highly predictive of aggressive disease and poor prognosis, although it is worth noting that only disability progression was significantly different.

The presence of B cells and their secreted antibody and FLC in the CSF and brain tissue is well established.^10 17 20 26 27^ However, little evidence exists for a direct role of secreted antibody in MS pathogenesis. The success of anti-B cell therapy points towards this,^28 29^ but cannot be distinguished from the role of B cells as highly efficient presenters of antigen to T cells. Our data raise an intriguing possibility that the spreading of the antibody repertoire, starting from the predominant use of κ light chains and leading to a mixture of κ and λ, might be involved in disease progression and therefore pathogenesis, with remaining questions about the antigen specificity of these antibodies. There are many candidate antigens in the literature,^30–33^ and it is possible that some of these account for the differential light chain usage. Although most research focuses on the role of heavy chains in antigen recognition, accumulation of hypermutated sequences in the complementarity-determining regions and the codominant role of heavy and light chains in antigen binding demonstrate a clear role for light chains.^34 35^

The availability of a prognostic marker in the CSF at the time of diagnosis is of clear utility. There are now many highly efficacious therapies for MS, making a rational choice difficult for any individual patient, particularly as some are associated with a significant adverse event profile. MRI protocols are now well established for the diagnosis and therapeutic monitoring of patients with MS. The use of CSF κ and λ assay measurements at diagnosis may help to identify patients with a poor prognosis, justifying the use of highly effective therapies, with potential benefit on long-term outcomes. Larger independent studies would be required to validate the findings and determine the ultimate clinical utility of these measurements.
