# Supplementary material for: Cerebrospinal fluid immunoglobulin light chain ratios predict disease progression in multiple sclerosis
Source: J Neurol Neurosurg Psychiatry. 2018 May 9;89(10):1044–9. doi: 10.1136/jnnp-2018-317947 (PMC6166608; doi:10.1136/jnnp-2018-317947)
Supplement: Supplementary file 1 [file jnnp-2018-317947supp001.pdf]

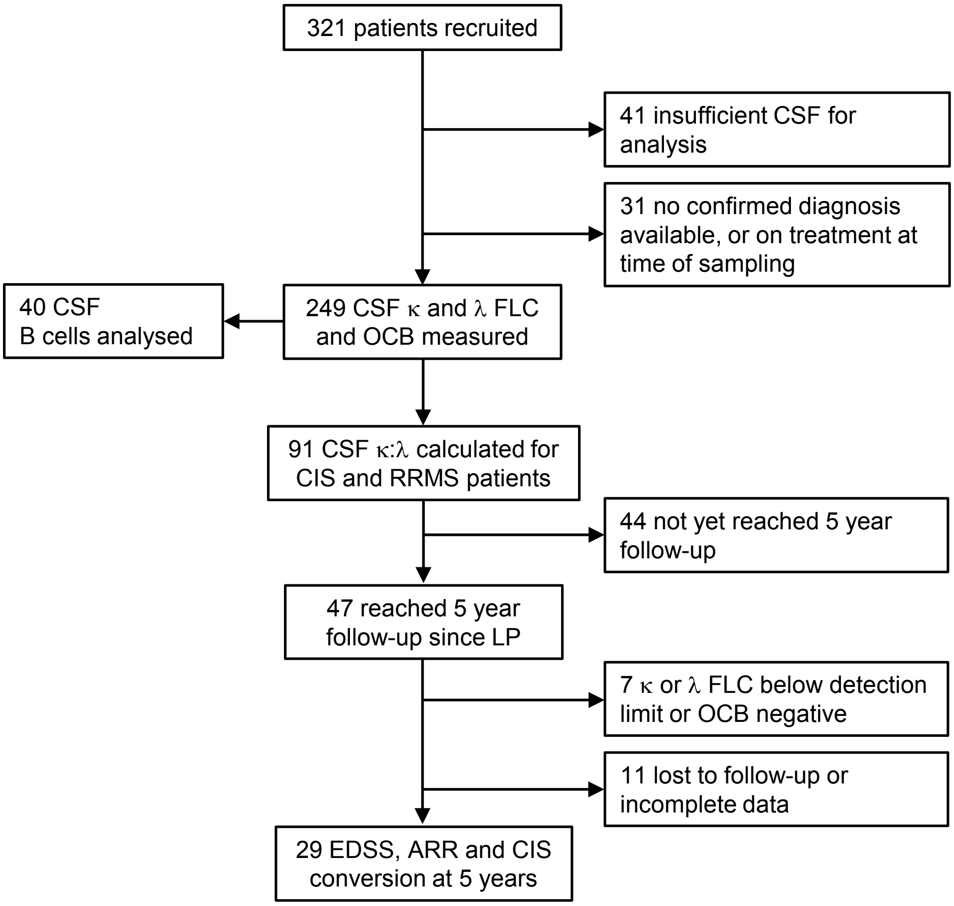

Figure e1. Study design.

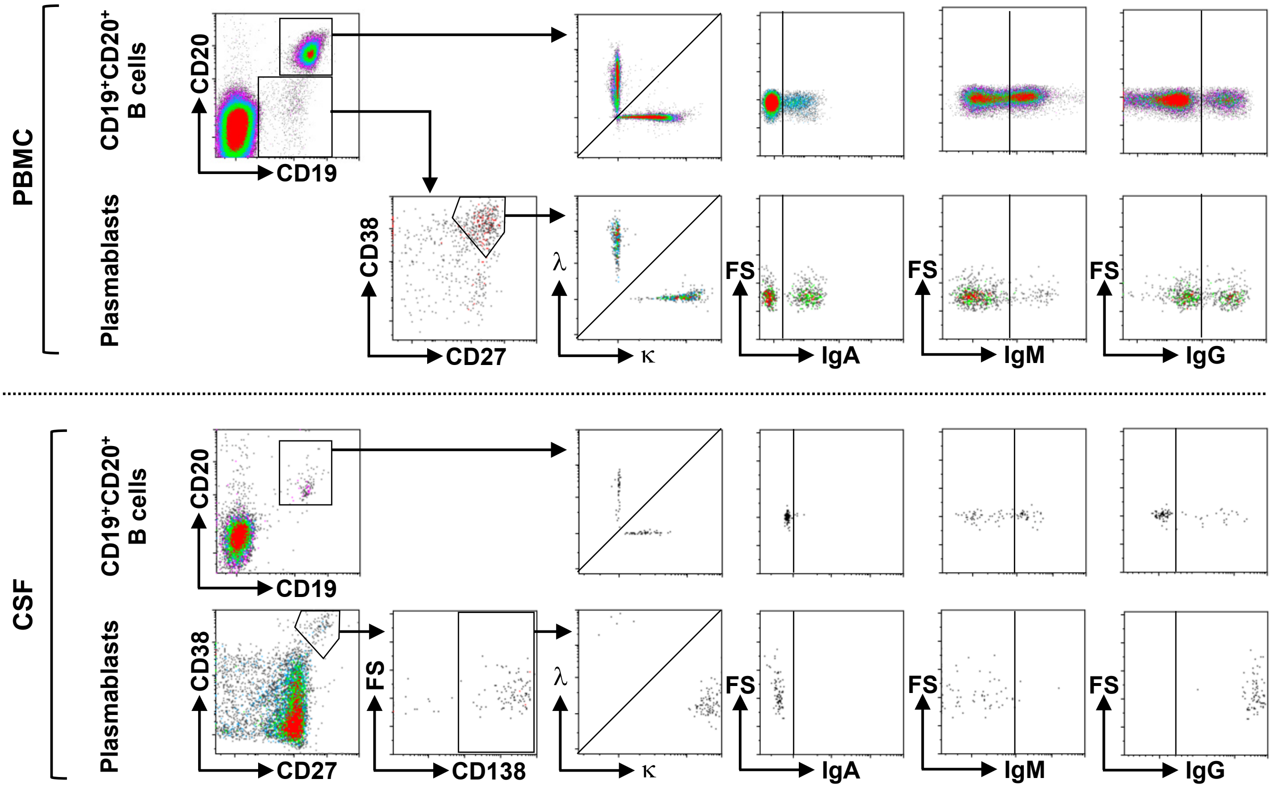

Figure e2. Staining and gating strategy for detection of B cells and plasmablasts. Peripheral blood mononuclear cells (PBMC) and cerebrospinal fluid (CSF) cells were stained with Zombie Aqua™ Fixable Viability dye in PBS (BioLegend, London, UK) at 4ºC for 20 min and washed in PBS 2% BSA (Sigma-Aldrich) at 300 *g* for 4 min. Cells were stained for 20 mins at 4ºC with fluorescently labelled antibodies to detect surface markers, fixed and permeabilised (Foxp3/transcription factor buffer kit eBioscience, Hatfield, UK) and stained for 20 min to detect intracellular markers. Cells were analysed on a BD LSR Fortessa™ flow cytometer (BD Biosciences, Oxford, UK) and data analysed using Kaluza Flow Cytometry Analysis Software v1.2 (Beckman Coulter, High Wycombe, UK). The following monoclonal antibodies were used (Biolegend unless stated otherwise); anti-CD19 Bv605 (HIB19), -CD20 Bv711 (2H7), -CD27 APC-Cy7 (O323), -CD138 PE (MI15), -CD38 PE-Cy7 (HB-7), -Ig kappa PerCP-Cy5.5 (MHK-49), -Ig lambda APC (MHL-38), -IgA FITC (IS11-8E10; Miltenyi Biotec), -IgG PE-CF594 (G18-145; BD Biosciences) and -IgM Bv650 (MHM-88). B cells were defined as $CD19^{+}CD20^{+}$, with blood plasmablasts identified as $CD27^{high}CD38^{high}$ within the $CD19^{+}$ gate, and CSF plasmablasts defined as $CD27^{high}CD38^{high}CD138^{high}$. FS; forward scatter.
